# Supplementary material for: The influence of fetal sex on maternal blood pressure in pregnancy
Source: medRxiv. 2025 Jan 29:2025.01.28.25321287. Preprint. [Version 1] doi: 10.1101/2025.01.28.25321287 (PMC11839000; doi:10.1101/2025.01.28.25321287)
Supplement: Supplement 1 [file media-1.pdf]

S1 Table. 186 autosomal own-birth-weight-associated lead SNPs, oriented to the increaser allele. From the marginal analysis of Warrington et al. 2019 genome-wide association study for birth weight.

SNP	Chrom:position (hg19)	Trait-raising Allele	Trait-lowering Allele	Beta (SEM[a] adjusted fetal effects)
rs17367504	chr1:11862778	A	G	0.005
rs12401656	chr1:43456767	G	A	0.029
rs80278614	chr1:119412317	A	G	0.052
rs905938	chr1:154991389	C	T	0.023
rs670523	chr1:155878732	G	A	0.016
rs72480273	chr1:161644871	C	A	0.022
rs10913200	chr1:176521655	G	A	0.038
rs61830764	chr1:212289976	A	G	0.018
rs3806315	chr1:214724668	A	G	0.016
rs708122	chr1:228216997	C	A	0.015
rs10495563	chr2:9662210	A	G	0.016
rs2551347	chr2:23912401	T	C	0.029
rs1179494	chr2:36809496	G	C	0.002
rs754868	chr2:43185532	G	A	0.019
rs4952673	chr2:43423870	G	A	0.004
rs17034876	chr2:46484310	T	C	0.039
rs4953353	chr2:46567276	G	T	0.019
rs186606513	chr2:97482001	G	A	0.047
rs56188432	chr2:158406865	G	A	0.25
rs560887	chr2:169763148	T	C	0.025
rs2280235	chr2:191843830	G	A	0.014
rs10181515	chr2:227019461	T	C	0.021
rs9855896	chr3:14287150	A	G	0.014
rs2168443	chr3:46947087	T	A	0.01
rs11708067	chr3:123065778	G	A	0.056
rs9851257	chr3:123125711	T	A	0.005
rs6440006	chr3:141142691	G	A	0.001
rs2306700	chr3:142123841	T	C	0.022
rs10935733	chr3:148622968	T	C	0.021
rs4679760	chr3:155855418	C	G	0.009
rs1482852	chr3:156798294	A	G	0.054
rs11711420	chr3:183349010	T	G	0.022
rs4144829	chr4:17903654	C	T	0.032
rs6533183	chr4:106133184	C	T	0.008
rs116807401	chr4:135121721	C	T	0.088
rs6845999	chr4:145565826	T	C	0.017
rs4579095	chr4:174726635	A	G	0.007
rs1818782	chr5:39424628	C	A	0.015
rs351930	chr5:52003397	T	A	0.02
rs854037	chr5:57091783	A	G	0.02
rs28365970	chr5:67585723	C	A	0.015

SNP	Chrom:position (hg19)	Trait-raising Allele	Trait-lowering Allele	Beta (SEM[a] adjusted fetal effects)
rs1981627	chr5:133838180	G	A	0.007
rs2946179	chr5:157886627	T	C	0.004
rs34471628	chr5:172196752	G	A	0.014
rs9379084	chr6:7231843	G	A	0.004
rs35261542	chr6:20675792	C	A	0.049
rs9379832	chr6:26186200	A	G	0.019
rs9366778	chr6:31269173	G	A	0.014
rs6911024	chr6:31368451	C	T	0.002
rs9267812	chr6:32128394	T	C	0.015
rs1547669	chr6:33775641	G	A	0.018
rs75104038	chr6:34190104	A	G	0.024
rs9348981	chr6:35687249	T	G	0.015
rs7744700	chr6:53349401	T	A	0.018
rs76094073	chr6:109288036	G	C	0.011
rs6925689	chr6:126865884	T	C	0.018
rs6569647	chr6:130337266	T	C	0.014
rs6930558	chr6:141878920	T	G	0.022
rs962554	chr6:142734204	T	C	0.015
rs10872678	chr6:152039964	T	C	0.028
rs2934844	chr6:166142456	T	A	0.018
rs4719648	chr7:2756832	C	T	0.014
rs59084784	chr7:22739562	A	C	0.011
rs7808457	chr7:22798265	A	T	0.002
rs34776209	chr7:23513093	C	T	0.015
rs2908279	chr7:44174857	T	G	0.007
rs2971669	chr7:44231778	C	T	0.003
rs138715366	chr7:44246271	C	T	0.235
rs10265133	chr7:45895604	T	G	0.02
rs11983722	chr7:46298647	A	T	0.029
rs10265057	chr7:47275737	G	A	0.036
rs2237467	chr7:50733316	A	G	0.011
rs112139215	chr7:73034559	A	C	0.056
rs2282978	chr7:92264410	C	T	0.021
rs45446698	chr7:99332948	T	G	0.017
rs6467157	chr7:127660763	T	C	0.014
rs3918226	chr7:150690176	T	C	0.005
rs62496903	chr8:6446938	T	C	0.028
rs732563	chr8:23345526	C	T	0.019
rs11778247	chr8:23403378	A	G	0
rs34036147	chr8:38366249	T	C	0.019
rs13266210	chr8:41533514	A	G	0.03
rs72656010	chr8:57122215	T	C	0.026
rs6995390	chr8:77611012	A	T	0.014
rs7819593	chr8:106115172	C	T	0.023
rs10283100	chr8:120596023	G	A	0.033

SNP	Chrom:position (hg19)	Trait-raising Allele	Trait-lowering Allele	Beta (SEM[a] adjusted fetal effects)
rs13271368	chr8:126506140	C	T	0.021
rs13257363	chr8:142252580	G	A	0.017
rs9657468	chr8:142362391	G	T	0.018
rs7854962	chr9:96900505	C	G	0.016
rs28457693	chr9:98217348	G	A	0.04
rs2418135	chr9:113901309	A	G	0.012
rs72760655	chr9:116916214	A	C	0.009
rs1323438	chr9:119115531	C	T	0.02
rs3933326	chr9:123633948	G	A	0.023
rs10985827	chr9:125701608	G	T	0.027
rs28505901	chr9:139241030	A	G	0.024
rs4350272	chr10:25056118	A	G	0.017
rs9645500	chr10:70986723	G	T	0.019
rs1112718	chr10:94479107	G	A	0.036
rs10509669	chr10:95969913	T	A	0.02
rs3740360	chr10:96025491	C	A	0.003
rs2274224	chr10:96039597	C	G	0.019
rs562974282	chr10:104201070	T	G	0.126
rs10883846	chr10:104958244	C	T	0.016
rs7903146	chr10:114758349	T	C	0.003
rs7076938	chr10:115789375	T	C	0.029
rs71486610	chr10:124134803	C	G	0.016
rs11042596	chr11:2118860	T	G	0.027
rs234864	chr11:2857297	A	G	0.017
rs2168101	chr11:8255408	A	C	0.015
rs4444073	chr11:10331664	A	C	0.023
rs5030317	chr11:32410337	C	G	0.007
rs10437653	chr11:46297631	A	C	0.002
rs10734564	chr11:48160429	G	A	0.009
rs667515	chr11:69449076	G	C	0.013
rs61885091	chr11:69791952	A	G	0.024
rs10830963	chr11:92708710	C	G	0.002
rs10895278	chr11:102095335	T	C	0.001
rs76895963	chr12:4384844	G	T	0.051
rs11055030	chr12:12878349	G	C	0.022
rs2306547	chr12:26877885	C	T	0.016
rs11051061	chr12:30914668	A	G	0.001
rs6582623	chr12:46613394	C	T	0.02
rs180438	chr12:47187260	A	G	0.007
rs7968682	chr12:66371880	G	T	0.037
rs1480470	chr12:66412130	G	A	0.028
rs1533688	chr12:102772745	T	C	0.004
rs2647873	chr12:103081192	A	G	0.009
rs17033114	chr12:103123339	C	T	0.008
rs3184504	chr12:111884608	C	T	0.005

SNP	Chrom:position (hg19)	Trait-raising Allele	Trait-lowering Allele	Beta (SEM[a] adjusted fetal effects)
rs9549046	chr13:40647206	A	G	0.027
rs34217484	chr13:48854550	A	T	0.012
rs9318511	chr13:78601413	C	A	0.024
rs72681869	chr14:50655357	C	G	0.108
rs6575803	chr14:101257755	C	T	0.034
rs75844534	chr15:38667117	A	C	0.036
rs2928148	chr15:41401550	G	A	0.004
rs339969	chr15:60883281	A	C	0.011
rs3784789	chr15:75082552	C	G	0.018
rs12909648	chr15:86224570	A	G	0.003
rs12443252	chr15:91064690	C	T	0.007
rs4932373	chr15:91429287	A	C	0.01
rs55958435	chr15:96852638	A	G	0.022
rs7402983	chr15:99193276	A	C	0.027
rs11630479	chr15:99240481	G	A	0.007
rs2045457	chr16:20046115	G	A	0.012
rs40434	chr16:55699525	G	A	0.017
rs28544888	chr16:55741204	C	T	0.027
rs11641308	chr16:75312023	C	T	0.005
rs222857	chr17:7164563	T	C	0.026
rs4511593	chr17:7455536	T	C	0.019
rs78378222	chr17:7571752	G	T	0.058
rs9909342	chr17:25652275	A	G	0.019
rs7223535	chr17:29211667	G	A	0.02
rs11867479	chr17:68090207	T	C	0.018
rs10221267	chr17:68464662	T	C	0.018
rs73354194	chr17:79905947	C	T	0.06
rs9912553	chr17:79959703	G	C	0.006
rs11082304	chr18:20720973	T	G	0.013
rs2779165	chr19:4915447	G	C	0.018
rs8106042	chr19:7161849	G	C	0.023
rs2967676	chr19:8789666	C	A	0.003
rs41355649	chr19:33790556	G	A	0.042
rs1129156	chr19:40719076	T	C	0.022
rs147957154	chr19:43431040	T	C	0.026
rs516246	chr19:49206172	C	T	0.017
rs255773	chr19:54723546	C	T	0.018
rs147110934	chr19:55993436	G	T	0.055
rs12461110	chr19:56320663	G	A	0.005
rs304001	chr19:56423668	A	G	0.003
rs6040076	chr20:10658882	C	G	0.015
rs6033062	chr20:11207419	A	T	0.014
rs1203876	chr20:22540915	C	A	0.055
rs11698914	chr20:31327144	C	G	0.029
rs181451002	chr20:32466219	A	G	0.006

SNP	Chrom:position (hg19)	Trait-raising Allele	Trait-lowering Allele	Beta (SEM[a] adjusted fetal effects)
rs2889874	chr20:33715777	G	T	0.014
rs1012167	chr20:39159119	C	T	0.024
rs753381	chr20:39797465	T	C	0.018
rs6026449	chr20:57272617	C	T	0.018
rs73143584	chr20:62445702	A	G	0.031
rs2229742	chr21:16339172	G	C	0.028
rs220193	chr21:43581308	A	G	0.018
rs134594	chr22:29468456	C	T	0.022
rs41311445	chr22:42070374	A	C	0.034
rs7285579	chr22:46441980	C	T	0.018

[a]SEM = structural equation model for partitioning of maternal and fetal effects. Note: genetic scores were unweighted i.e. all SNP weights set to 1 for analysis
